# Calculation of Transient Potential Rise on the Wind Turbine Struck by Lightning

**DOI:** 10.1155/2014/213541

**Published:** 2014-08-31

**Authors:** Zhang Xiaoqing

**Affiliations:** National Active Distribution Network Technology Research Center, School of Electrical Engineering, Beijing Jiaotong University, Beijing 100044, China

## Abstract

A circuit model is proposed in this paper for calculating the transient potential rise on the wind turbine struck by lightning. The model integrates the blade, sliding contact site, and tower and grounding system of the wind turbine into an equivalent circuit. The lightning current path from the attachment point to the ground can be fully described by the equivalent circuit. The transient potential responses are obtained in the different positions on the wind turbine by solving the circuit equations. In order to check the validity of the model, the laboratory measurement is made with a reduced-scale wind turbine. The measured potential waveform is compared with the calculated one and a better agreement is shown between them. The practical applicability of the model is also examined by a numerical example of a 2 MW Chinese-built wind turbine.

## 1. Introduction

As wind power generation undergoes rapid growth, wind turbines (WTs) erected in large wind farms are regarded as a realistic alternative to conventional power plants. Due to their large height of towers, long rotating blades, and exposed locations, WTs can be easily struck by lightning. When a WT suffers a lightning stroke, a large lightning current will continue to flow from the attachment point to the grounding system and cause the transient potential rise on the WT. The high potential can sparkover the insulation distance and severely damage the WT components inside the structure. It can also give rise to the overvoltage surge that endangers the sensitive electronics and microprocessors in the WT control system. In the light of the serious problem arising from the transient potential rise, the protection design for the potential rise phenomenon has been paid more and more attention. In the protection design, the need exists for calculating the transient potential responses on the WT under lightning stroke. A few calculation methods were reported in literature [1–3]. In the previous methods, the tower, that is, the longest traveling path of lightning current on the WT, was modeled as a uniform transmission line. They are simple and easy to be adopted in the lightning transient calculation but incapable of calculating the transient potential responses in the different positions on the WT owing to their neglecting of the structural feature of the tower body. Recently, a more detailed method has been presented. This method represents the tower as a cage-like multiconductor system [4, 5] and can give the potential distribution on the WT; however, it causes a significant increase in the circuit complexity. For an improvement on the lightning transient calculation of WTs, a simplified circuit model is proposed in this paper. The proposed model divides the tower into a series of hollow cylindrical sections and represents it as a *π*-type circuit chain instead of the complicated cage-like multiconductor system. Based on the simplified treatment for the tower, a WT is converted into an equivalent circuit that can give a complete description of lightning current path including the blade, sliding contact site, and tower and grounding system. Then, transient potential responses are obtained in the different positions on the WT by performing the transient calculation for the equivalent circuit. In order to confirm the validity of the proposed model, a laboratory experiment has also been carried out on a reduced-scale WT.

## 2. Circuit Parameters

### 2.1. Blade

**Figure 1:**
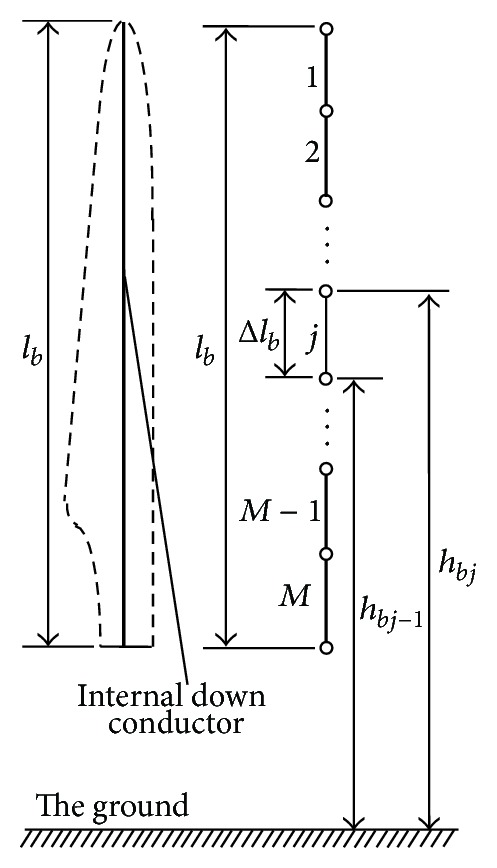
Segmentation of down conductor.

**Figure 2:**
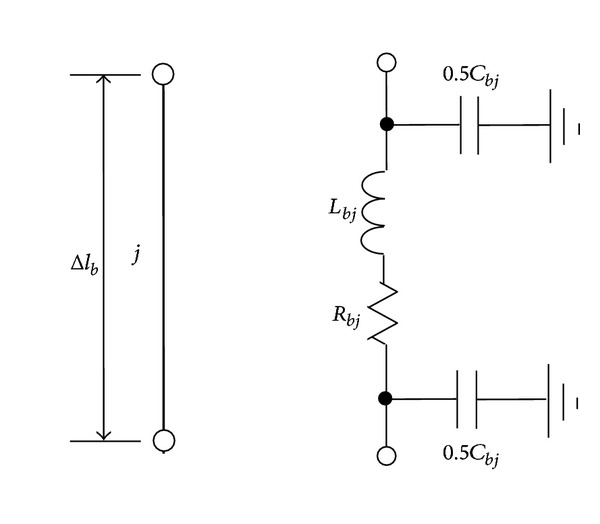
*π*-type circuit of a down conductor segment.

An internal down conductor is usually installed inside the blade for conducting the lightning current from the attachment point to the hub, as shown in Figure 1. To take account of the propagation phenomenon of lightning current, the down conductor is divided into a suitable number of segments. The segment length Δ*l*_*b*_ needs to fulfill the following criterion [6]:

(1) $$\begin{array}{c} \Delta l_{b}<\frac{1}{10}\frac{c}{f_{u}}, \end{array}$$

where *c* is the velocity of light and *f*_*u*_ the maximum frequency likely to affect the system transient. An arbitrary segment *j*  (*j* = 1,2,…, *M*) in Figure 1 can be represented by a *π*-type circuit composed of resistance, inductance, and capacitance, as shown in Figure 2. The resistance *R*_*bj*_ is estimated by [7]

(2) $$\begin{array}{c} R_{bj}=\frac{\sqrt{\mu_{b}f_{u}}}{2r_{b}\sqrt{\pi \sigma_{b}}}, \end{array}$$

where *μ*_*b*_ and *σ*_*b*_ are the material permeability and conductivity of the down conductor, respectively, and *r*_*b*_ is the conductor radius. The inductance *L*_*bj*_ is calculated by [5]

(3) $$\begin{array}{c} L_{bj}=\frac{\mu_{0}}{4\pi }\left(S_{j}+S_{j}^{\prime }\right), \end{array}$$

where *μ*_0_ is the permeability of free space (4*π* × 10^−7^ H/m) and the two parameters in the round brackets are

(4) $$\begin{array}{c} S_{j}=r_{b}\left[2+\varphi \left(\frac{h_{bj}-h_{bj-1}}{r_{b}}\right)+\varphi \left(-\frac{h_{bj}-h_{bj-1}}{r_{b}}\right)\right]\\ S_{j}^{\prime }=r_{b}\left[\varphi \left(-\frac{2h_{bj-1}}{r_{b}}\right)+\varphi \left(-\frac{2h_{bj}}{r_{b}}\right)-2\varphi \left(-\frac{h_{bj-1}+h_{bj}}{r_{b}}\right)\right], \end{array}$$

where $\varphi (\xi )=\xi \sinh^{-1}\xi -\sqrt{1+\xi^{2}}$. According to the electromagnetic analogy [8–10], the capacitance *C*_*bj*_ is determined by

(5) $$\begin{array}{c} C_{bj}=\frac{\mu_{0}\varepsilon_{0}}{L_{bj}}, \end{array}$$

where *ε*_0_ is the permittivity of free space [(36*π*)^−1^× 10^−9^ F/m].

### 2.2. Sliding Contact Site

**Figure 3:**
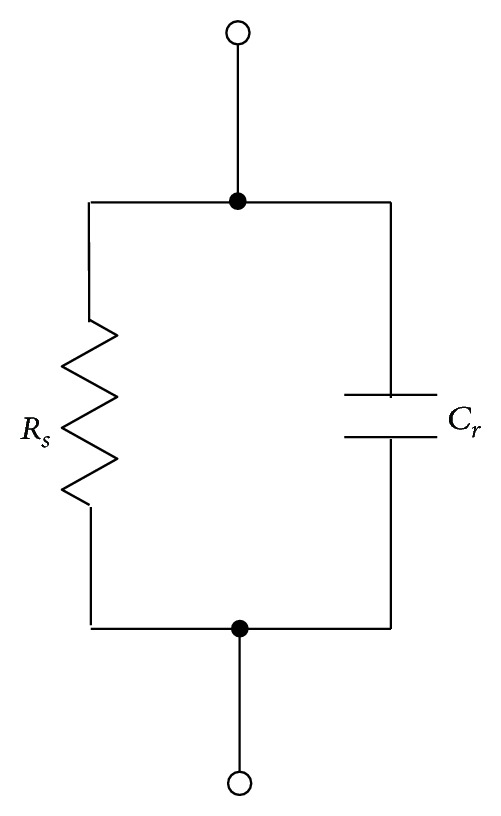
Parallel circuit of sliding contact site.

The conductive path between the blade root and tower top is a sliding contact site and mainly includes the brushes, sliding contact systems, and main shaft bearings. The brushes have been widely used in the multimegawatt WTs to divert the lightning current from the blade root to the tower top. The circuit parameters of the brushes and main shaft bearings are simply represented as the contact resistance *R*_*S*_ and equivalent capacitance *C*_*r*_ [3, 5], respectively. *C*_*r*_ can be evaluated by the formula given in [11]. Since the nacelle is rarely tuning, the yaw bearing is not considered here. In view of the actual shunting route of lightning current, the sliding contact site is modeled as a simple parallel circuit, as shown in Figure 3.

### 2.3. Tower and Grounding System

**Figure 4:**
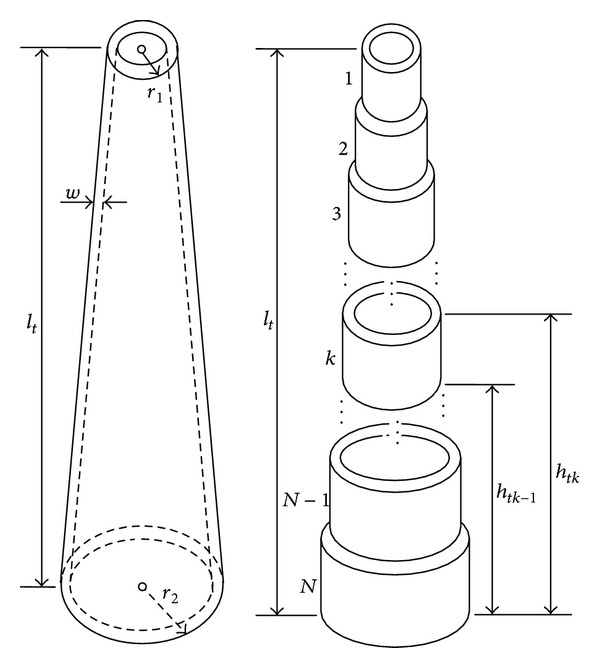
Approximate division of tower body.

**Figure 5:**
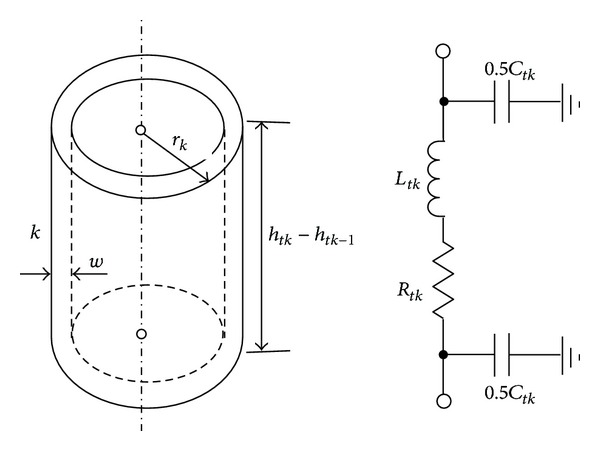
*π*-type circuit of a hollow cylinder section.

An actual tower takes the shape of the hollow circular truncated cone, as shown in Figure 4, and is the longest traveling path of lightning current. As is the case in the blade, consideration of the propagation phenomenon of the lightning current needs the tower to be divided into a certain number of sections. Each section is approximately taken as a hollow cylinder and its length is determined by (1). An arbitrary section *k* (*k* = 1,2,…, *N*) in Figure 4 can also be represented by a *π*-type circuit, as shown in Figure 5. The resistance *R*_*tk*_ is roughly evaluated by [12, 13]

(6) $$\begin{array}{c} R_{tk}=\left[1+\left(\sqrt{\frac{q}{2}}-1\right)\left(1-\frac{w}{r_{k}}-\frac{8}{4\sqrt{2q}-5}{\left(\frac{w}{r_{k}}\right)}^{2}\right)\right]R_{tk0}, \end{array}$$

where *q* = 2*πf*_*u*_ *μ*_*t*_ *σ*_*t*_ *w* ^2^ and *R*_*tk*0_ is the DC resistance

(7) $$\begin{array}{c} R_{tk0}=\frac{h_{tk}-h_{tk-1}}{\sigma_{t}\pi \left[r_{k}^{2}-{\left(r_{k}-w\right)}^{2}\right]}, \end{array}$$

where *μ*_*t*_ and *σ*_*t*_ are the material permeability and conductivity of the tower, respectively. The inductance *L*_*tk*_ is given by [14]

(8) $$\begin{array}{c} L_{tk}=\frac{\mu_{0}}{2\pi }\left[\ln\frac{2\left(h_{tk}-h_{tk-1}\right)}{r_{k}}-1-\frac{\mu_{t}}{\mu_{0}}\ln\eta \right], \end{array}$$

where *η* is the geometrical factor depending on the ratio of *p* = (*r*_*k*_ − *w*)/*r*_*k*_.  Table 1 gives the values of *η* in the range of *p* = 0 ~ 1. By substituting *L*_*tk*_ into (5), the capacitance *C*_*tk*_ can be obtained.

For the sake of simplification, the grounding system is modeled as a grounding resistance *R*_*g*_ [1, 4]. The value of *R*_*g*_ is specified by the corresponding design standard [15].

## 3. Circuit Model

**Figure 6:**
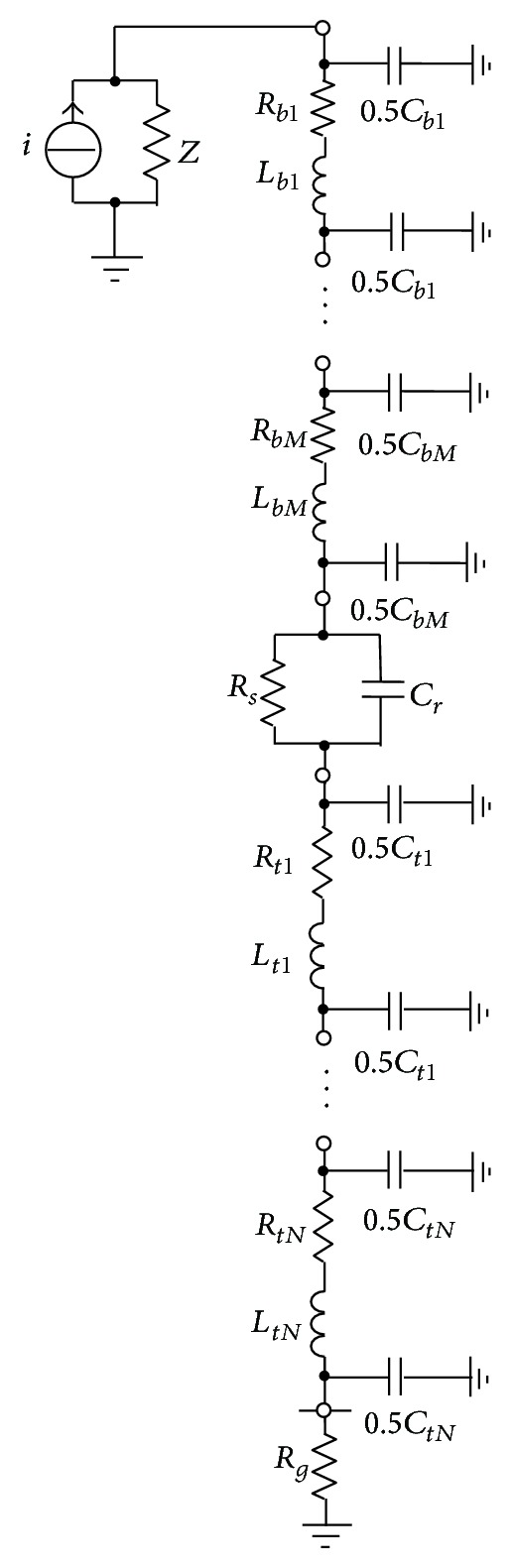
Complete equivalent circuit of a WT.

**Figure 7:**
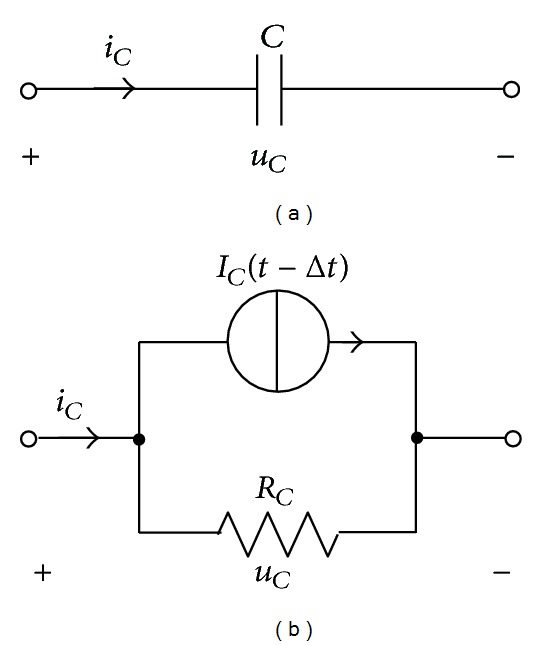
Discretization representation of capacitance. (a) Capacitance and (b) parallel circuit unit.

After obtaining the circuit parameters of the blade, sliding contact site, and tower and grounding system, a complete equivalent circuit can be built for a WT, as shown in Figure 6. The lightning current source *i* is injected to the top node of the equivalent circuit to simulate a lightning stroke to the blade tip and the impedance *Z* in parallel with *i* is the surge impedance of the lightning channel. For the capacitance in the equivalent circuit, as shown in Figure 7(a), its circuit equation is written as

(9) $$\begin{array}{c} i_{C}=C\frac{du_{C}}{dt}. \end{array}$$

Integration of (9) between [*t* − Δ*t*, *t*] gives

(10) $$\begin{array}{c} \overset{t}{\underset{t-\Delta t}{\int }}i_{C}dt=\overset{t}{\underset{t-\Delta t}{\int }}C\frac{du_{C}}{dt}, \end{array}$$

where Δ*t* is the time step size. By means of the trapezoidal rule, the integration is evaluated by

(11) $$\begin{array}{c} \frac{i_{C}\left(t-\Delta t\right)+i_{C}\left(t\right)}{2}\Delta t=C\left[u_{C}\left(t\right)+u_{C}\left(t-\Delta t\right)\right]. \end{array}$$

This can be rewritten as

(12) $$\begin{array}{c} i_{C}\left(t\right)=\frac{1}{R_{C}}u_{C}\left(t\right)+I_{C}\left(t-\Delta t\right), \end{array}$$

where

(13) $$\begin{array}{c} R_{C}=\frac{\Delta t}{2C},\\ I_{C}\left(t-\Delta t\right)=-\frac{u_{C}\left(t-\Delta t\right)}{R_{C}}-i_{C}\left(t-\Delta t\right). \end{array}$$

**Figure 8:**
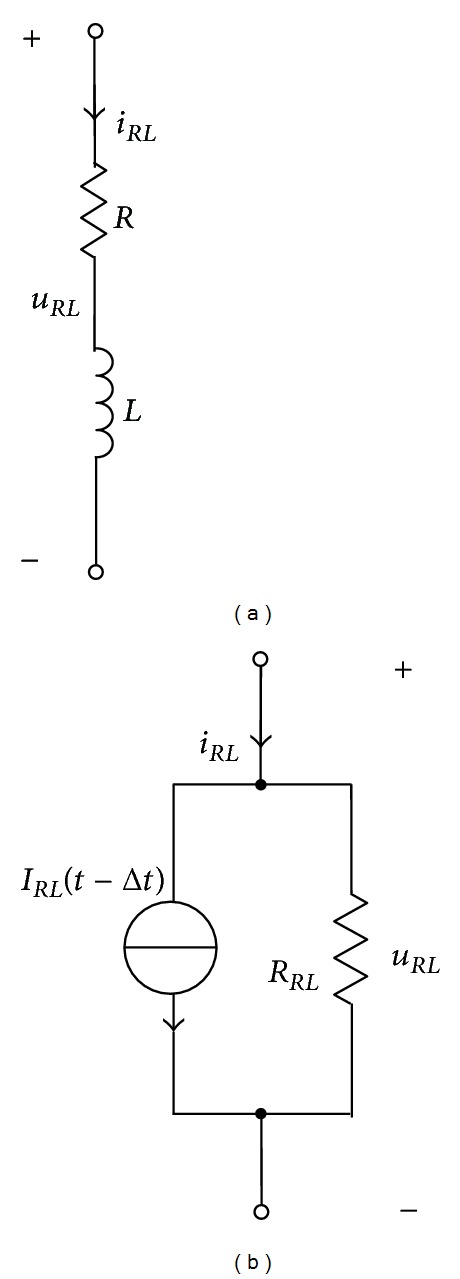
Discretization representation of *R*-*L* branch. (a) *R*-*L* branch and (b) parallel circuit unit.

The circuit model for (12) is a parallel circuit unit, as shown in Figure 7(b). For the resistance-inductance (*R*-*L*) branch in the equivalent circuit, as shown in Figure 8(a), its circuit equation is

(14) $$\begin{array}{c} u_{RL}=Ri_{RL}+L\frac{di_{RL}}{dt}; \end{array}$$

that is

(15) $$\begin{array}{c} \frac{di_{RL}}{dt}=\frac{1}{L}\left(u_{RL}-Ri_{RL}\right). \end{array}$$

Using the trapezoidal rule to integrate both sides of (15) between [*t* − Δ*t*, *t*] yields [16]

(16) iRL(t)−iRL(t−Δt)  =1LuRL(t)−RiRL(t)+uRL(t−Δt)−RiRL(t−Δt)2Δt.

Rearranging the terms in (16) leads to

(17) $$\begin{array}{c} i_{RL}\left(t\right)=\frac{1}{R_{RL}}u_{RL}\left(t\right)+I_{RL}\left(t-\Delta t\right), \end{array}$$

Where

(18) RRL=R+2LΔt,IRL(t−Δt)=1RRLuRL(t−Δt)+1RRL(RRL−2R)iRL(t−Δt).

**Figure 9:**
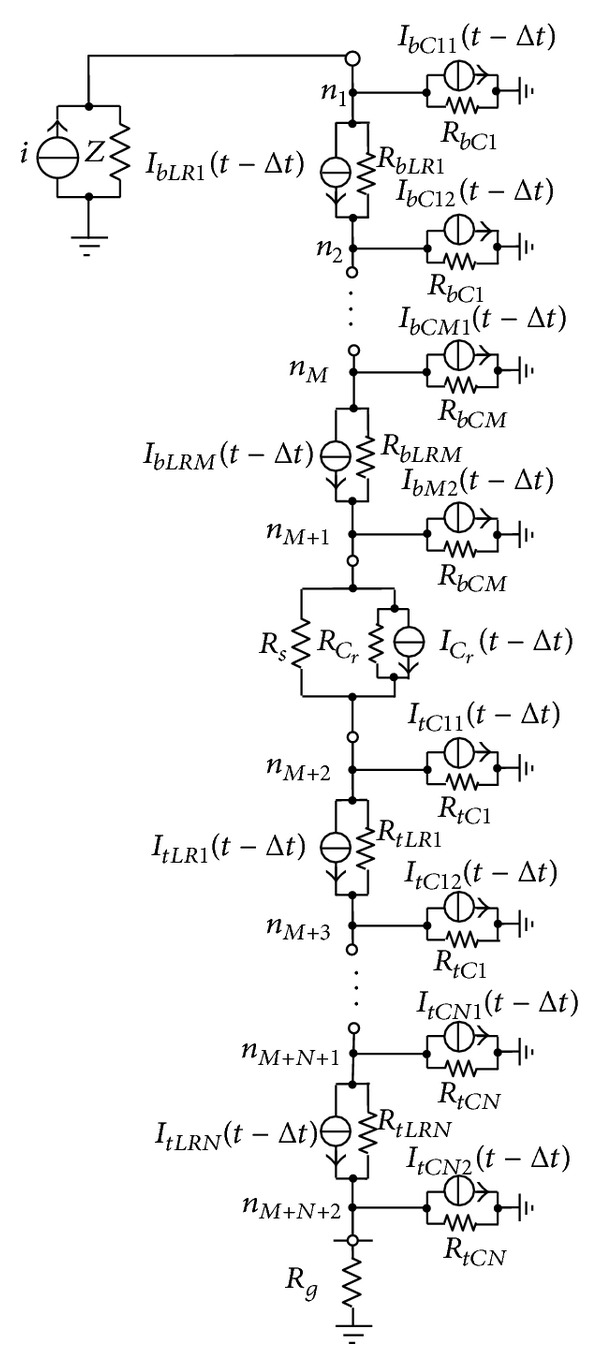
Time discretization circuit.

In terms of (17), the parallel circuit unit is depicted for the *R*-*L* branch, as shown in Figure 8(b). The current sources *I*_*C*_(*t* − Δ*t*) and *I*_*RL*_(*t* − Δ*t*) are known from the values for the preceding time step. After all capacitances and *R*-*L* branches are replaced by their respective parallel circuit units, Figure 6 is further converted into a time discretization circuit consisting only of resistances and current sources, as shown in Figure 9. The node voltage equations are set up for the time discretization circuit

(19) $$\begin{array}{c} G_{n}u_{n}=i_{n}, \end{array}$$

where **G**_*n*_ is the node conductance matrix, **u**_*n*_ the node voltage vector, and **i**_*n*_ the node current source vector. At *t* = 0, all capacitances and inductances are set in zero initial conditions. The node voltage vector **u**_*n*_  ([*u*_*n*1_,…,*u*_*nM*_,…,*u*_*nM*+*N*+2_]^*T*^) is obtained by solving (19) in each time step [17, 18]. Thus, the transient potential responses can be given for the different positions on the WT under lightning stroke.

## 4. Experimental Verification

**Figure 10:**
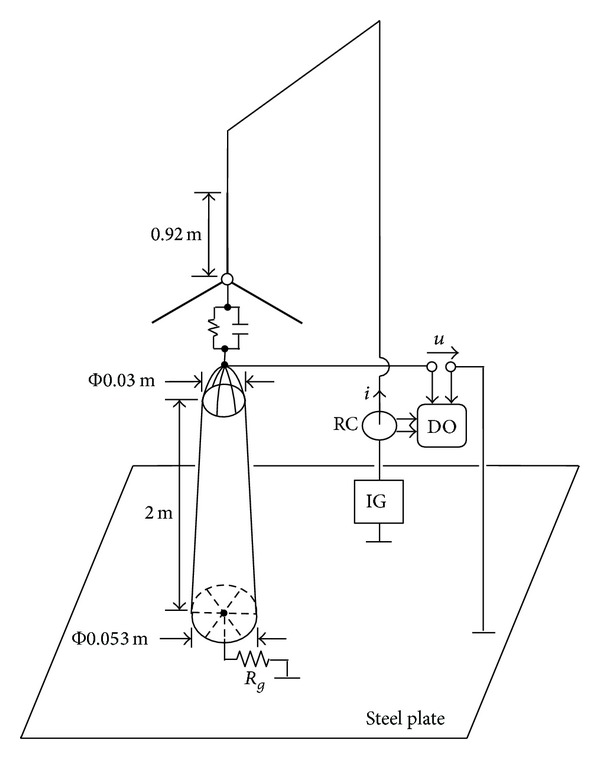
Experimental setup (RC: Rogowski coil; DO: digital oscilloscope; IG: impulse generator).

**Figure 11:**
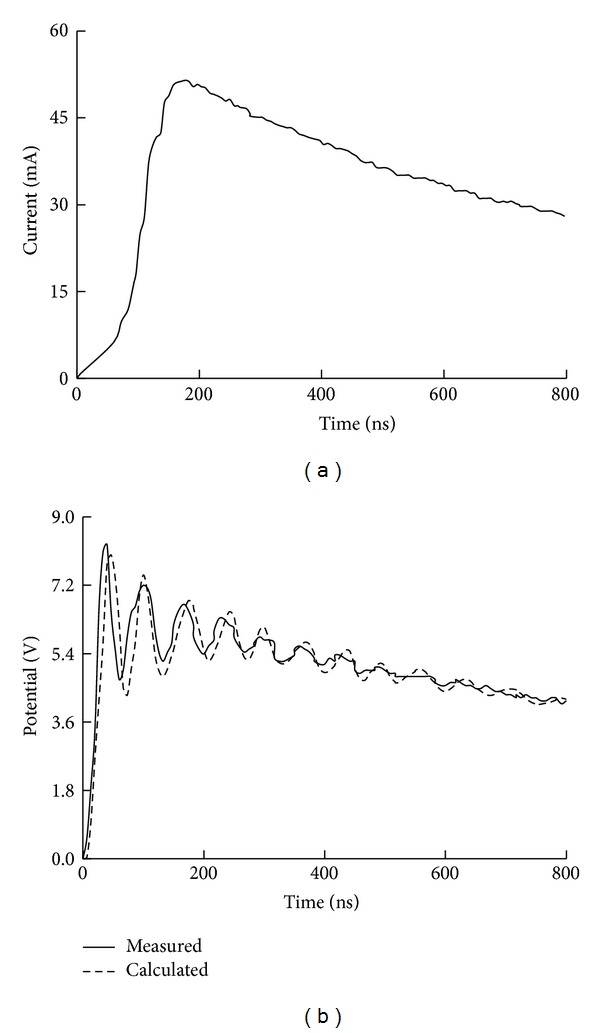
Comparison between measured and calculated waveforms. (a) Injected current. (b) Transient potentials at tower top.

A reduced-scale WT is built in the high voltage laboratory. Its dimensions are shown in Figure 10. The grounding resistance *R*_*g*_ is 4 Ω. The fast impulse current provided by an impulse generator is injected to the blade tip. The lead wire for measuring the transient potential is stretched perpendicular to the current lead wire and grounded (connected to the steel plate) at a point 8.0 m apart from the reduced-scale WT, which can restrain the electromagnetic induction between the two wires. In the experimental measurement, the current and potential signals are recorded by a digital oscilloscope. The measured waveforms of injected current and transient potential at the tower top are shown in Figure 11, where the corresponding waveform calculated from the circuit model proposed above is also given for comparison. It can be seen from Figure 11 that the calculated waveform is close to the measured one.

## 5. Calculated Results

**Figure 12:**
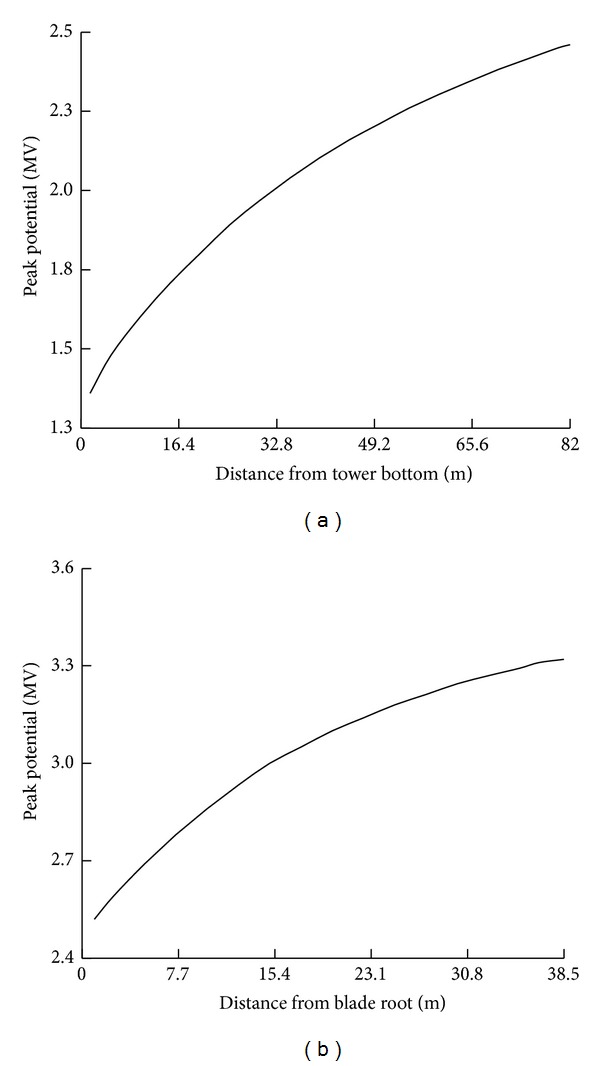
Peak potential distribution. (a) On tower. (b) On blade.

**Figure 13:**
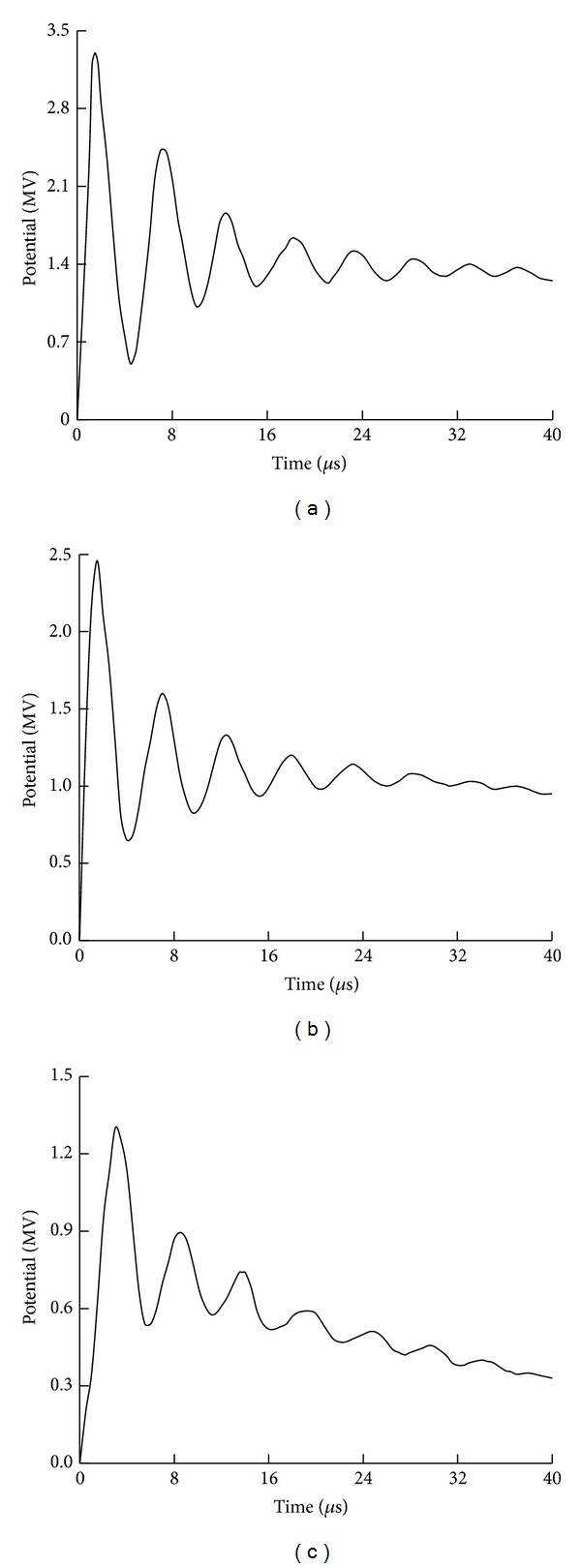
Transient potential waveforms. (a) At blade tip. (b) At tower top. (c) At tower bottom.

A 2MW Chinese-built WT is considered here. The dimensions of the WT are *l*_*b*_ = 38.5 m (see Figure 1), *l*_*t*_ = 82 m, *r*_1_ = 1.35 m, *r*_2_ = 2.16 m, and *w* = 0.025 m (see Figure 4). The grounding resistance *R*_*g*_ is 3 Ω and the lightning current *i* is taken as 10/350 *μ*s, 100 kA (see Figure 6) according to Chinese design standard [15, 19]. The peak potential distribution on the WT is plotted in Figure 12. The transient potential waveforms in three typical positions on the WT are also given in Figure 13. As seen from Figures 12 and 13, the transient potential rise on the WT is very serious and can do damage to the components and equipment inside the structure during a lightning stroke.

## 6. Conclusions

Calculation of the transient potential rise has been performed for the WT under lightning stroke. The lightning current path on the WT is described by a proposed circuit model that integrates the blade, sliding contact site, and tower and grounding system into a complete equivalent circuit. The model can predict the transient potential levels in the different positions on the WT. The calculated results obtained from the model indicate that the transient potential rise is serious and very harmful to the components inside the structure during a lightning stroke. The validity of the model has been confirmed by the laboratory experiment on a reduced-scale WT. The model is useful in lightning transient analysis of WTs and has the capability of providing a sound basis for the lightning protection design of WTs.

## Figures and Tables

**Table 1:** Values of geometrical factor *η*.

*P*	0	0.1	0.2	0.3	0.4	0.5
*η*	0.7788	0.7825	0.7930	0.8087	0.8286	0.8519

*P*	0.6	0.7	0.8	0.9	1.0	
*η*	0.8778	0.9058	0.9358	0.9672	1.000	
